# Conventional hybrid coronary vs. robot-assisted minimally invasive direct revascularization: a meta-analysis and systematic review

**DOI:** 10.3389/fcvm.2025.1650138

**Published:** 2025-12-01

**Authors:** Carla L. Schuering, Vanessa I. T. Zwaans, Anna Huang, Melanie Köhler-Seuster, Johanna K. R. von Mackensen, Jasper Iske, Julia Stein, Julius Kaemmel, Roland Heck, Christoph T. Starck, Jörg Kempfert, Stephan Jacobs, Volkmar Falk, Leonhard Wert

**Affiliations:** 1 Department of Cardiothoracic and Vascular Surgery, Deutsches Herzzentrum der Charité (DHZC), Berlin, Germany; 2 DZHK (German Center for Cardiovascular Research), Berlin, Germany; 3 Department of Cardiothoracic Surgery, Charité−Universitätsmedizin Berlin, Corporate Member of Freie Universität Berlin and Humboldt-Universität zu Berlin, Berlin, Germany; 4 Department of Health Sciences and Technology, ETH Zurich, Zurich, Switzerland

**Keywords:** hybrid coronary revascularisation, MIDCAB, PCI—percutaneous coronary intervention (PCI), hybrid, HCR, hybrid MIDCAB

## Abstract

**Objectives:**

Hybrid coronary revascularization (HCR), a revascularization strategy that amalgamates the minimally invasive direct coronary artery bypass (MIDCAB) procedure and percutaneous coronary intervention (PCI), represents a significant advancement in coronary artery disease treatment. This study compares conventional and robotic approaches in HCR.

**Methods:**

A systematic literature review and individual patient data analysis was conducted via PubMed following PRISMA guidelines, including original works published until 28 February 2025.

**Results:**

32 publications met the inclusion criteria, providing individual data from 2,048 patients. All patients underwent MIDCAB for LAD lesions and perioperative PCI for non-LAD lesions. 903 patients (670 male, 233 female; mean age 51.69 ± 7.77 years; BMI 34.66 ± 13.13) were treated with robot-assisted HCR, whilst 1,145 patients (890 male, 255 female; mean age 69.62 ± 8.42 years; BMI 26.62 ± 1.30) underwent conventional HCR. The robot-assisted group showed significantly higher rates of right coronary artery (RCA) stenosis (18.60% vs. 16.07%, *p* = 0.004) and drug-eluting stent use (62.68% vs. 5.42%, *p* = 0.027), along with significantly shorter hospital stays (4.27 ± 1.34 vs. 10.27 ± 7.34 days, *p* = 0.001). Although not statistically significant, wound complications were more frequent in the robot-assisted cohort (0.66% vs. 0.09%), whereas pleural effusion (10.74% vs. 0.00%), pericardial effusion (0.61% vs. 0.11%), and pneumothorax (1.83% vs. 0.11%) were more frequent in the conventional group. Mortality was low in both cohorts (robotic vs. conventional): intra-operative (0% vs. 0.09%, *p* = 0.73), 30-day (0.44% vs. 0.70%, *p* = 0.82), follow-up (2.66% vs. 4.72%, *p* = 0.41).

**Conclusion:**

Hybrid coronary revascularization offers a less invasive alternative with potential benefits. Robotic assistance may enhance outcomes, but limited adoption and heterogeneous data underscore the need for further investigation and validation.

## 1 Introduction

Coronary artery disease (CAD) remains a leading global cause of mortality, necessitating continuous advancements in revascularization strategies. Alongside optimal medical treatment and preventive measures, the primary therapeutic strategies for enhancing survival and quality of life for CAD patients include percutaneous coronary intervention (PCI) with drug-eluting stents (DES) and coronary artery bypass grafting (CABG) (1, 2). Hybrid coronary revascularization (HCR) has recently emerged as a promising approach that integrates minimally invasive direct coronary artery bypass (MIDCAB) for the left anterior descending (LAD) artery with PCI for non-LAD lesions.

Historically, HCR was reserved for high-risk patients unable to tolerate CABG. However, recent advancements in surgical and interventional techniques have expanded its applicability, particularly for patients with multivessel disease in whom the LAD is best treated surgically and other lesions by PCI. HCR exemplifies a patient-centered approach, providing tailored treatment based on coronary anatomy and comorbidities (3).

MIDCAB avoids major surgical trauma, including full sternotomy, aortic manipulation, and cardiopulmonary bypass, while reducing complications such as bleeding, transfusions, and infections (4). LIMA grafts for LAD lesions demonstrate over 95% 10-year graft patency, reinforcing the longevity and efficacy of this approach (5, 6).

PCI provides immediate symptom relief and reduced recovery times, often allowing same-day discharge (7). DES have shown a restenosis rate of 5.2% at 6–12 months measured by target lesion revascularization (8). In contrast, saphenous vein grafts (SVG) exhibit higher stenosis rates, with a 12-month failure rate (defined as >75% stenosis) of 25.5%, as reported by Connie N. Hess et al., aligning with previous studies reporting 10%–20% failure rates (9). A recent review emphasized SVG failure variability: 11%–41% at <3 years, 19%–33% at 5–10 years, and 39%–61% at >10 years (10).

The reduced trauma of MIDCAB and minimal invasiveness of PCI align with contemporary preferences for less invasive procedures and quicker recovery (11). Mohr et al. (2013) found that in three-vessel disease, HCR yields comparable outcomes to conventional methods with the added benefits of minimally invasive surgery (12). Abdallah et al. (2013) similarly noted that HCR improves quality of life in multivessel CAD compared to traditional CABG (13).

Shroyer et al. and Stables et al. support the economic viability of HCR, especially relevant for healthcare systems burdened by chronic disease costs (14, 15).

Advancements in robotic and endoscopic techniques have further refined HCR by providing high-definition, three-dimensional imaging and superior dexterity for precise, stable maneuvers, facilitating access to challenging anatomy with reduced tissue trauma (16). These technologies improve visualization and instrument articulation, potentially reducing operative times and improving outcomes (17). Puskas et al. demonstrated that robot-assisted MIDCAB can be performed with high success rates and minimal complications (18).

To recognize this evolving technique, we conducted a systematic review and meta-analysis to assess the impact of robotic assistance on procedural efficacy and outcomes.

## 2 Materials and methods

This review was conducted in alignment with the Preferred Reporting Items for Systematic Reviews and Meta-Analyses (PRISMA) guidelines (19). The concept, inclusion criteria, research question and hypothesis were defined prior to the literature review. The objective was to compare conventional hybrid coronary and robot-assisted minimally invasive direct revascularization, based on the underlying hypothesis that robotic assistance would result in superior patient outcomes and enhanced survival rates.

### 2.1 Search strategies

**Figure 1:**
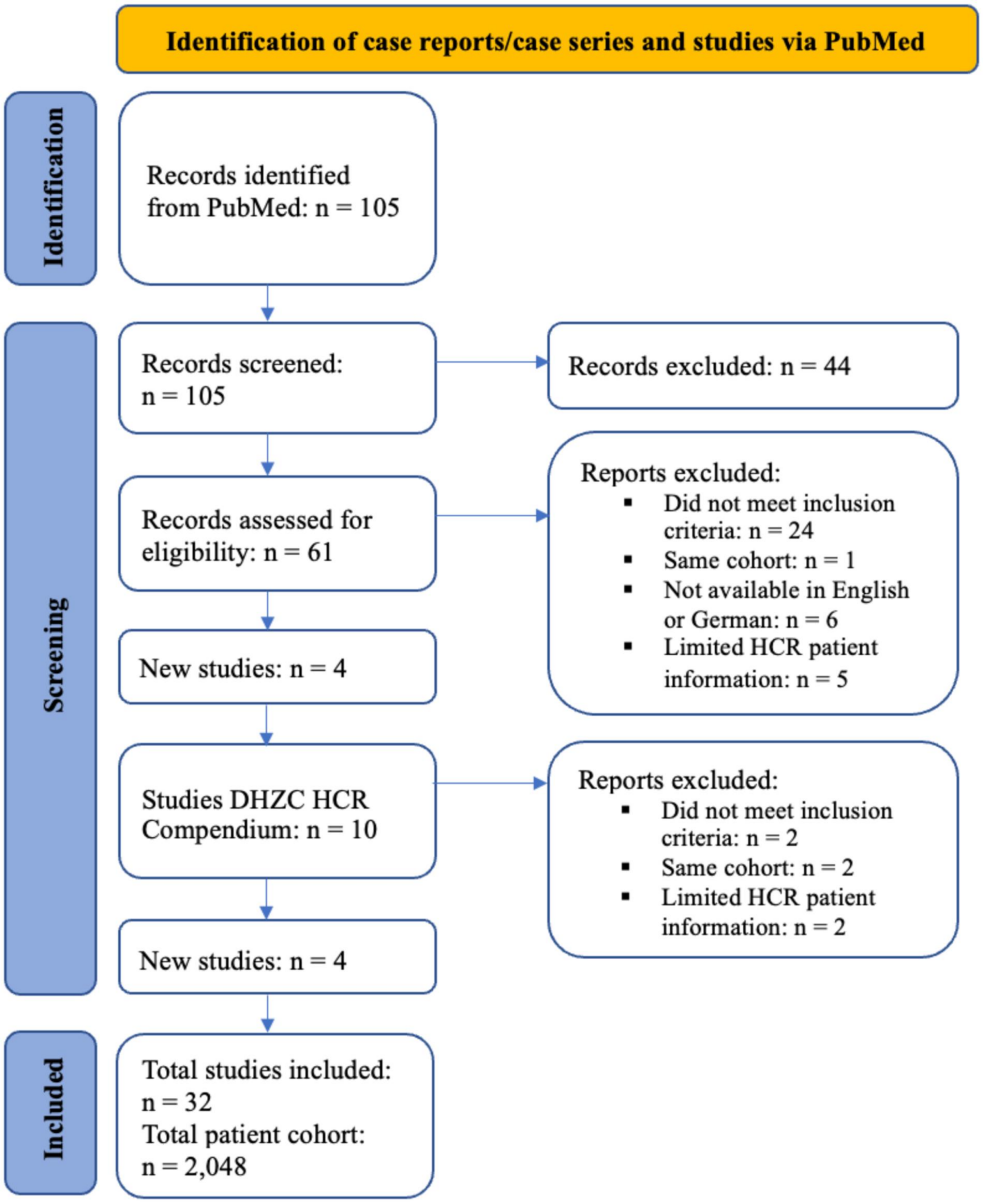
PRISMA flowchart of the systematic literature review.

A systematic PubMed search was conducted for articles published up to 28 February 2025 employing the terms: (“hybrid coronary revascularization” or “hybrid” or “HCR” or “PCI”) AND (“MIDCAB” or “RA-MIDCAB” or “RE-MIDCAB” or “TE-CAB” or “EACAB”). The titles of retrieved articles were scrutinized for relevance and duplicates were removed. Publications documenting hybrid coronary revascularization involving both MIDCAB and PCI techniques, were selected for further analysis. Full texts of all eligible articles were retrieved and reviewed by the first author (Figure 1).

### 2.2 Inclusion criteria

Included were case reports, case series, and retrospective, observational, or randomized studies involving CAD patients undergoing HCR. Eligibility required:

1. HCR involving both MIDCAB and PCI
2. LIMA grafting via MIDCAB for LAD lesions and non-LAD lesions suitable for PCI
3. Reported patient data (individual or for the relative HCR cohort as a whole) in terms of pre-intervention status, in-hospital short-term conditions and long-term outcomes.

Exclusion criteria comprised studies not published in English or German, those with inadequately detailed case descriptions and review articles. Case series and studies lacking primary data or those presenting pooled analysis without specific data on HCR patients were omitted.

### 2.3 Data extraction

Extracted variables included authorship, title, publication year, demographics (age, sex, body mass index), cardiac function (NYHA and/or CCS classifications, ejection fraction), comorbidities (diabetes, hypertension, dyslipidemia/hypercholesterolemia, smoking habits, family history, prior myocardial infarction). When coronary angiography was documented, detailed coronary anatomy was noted.

Intraoperative data included details on the revascularization procedure (e.g., MIDCAB, robotic assistance, off-pump, timing of PCI), operation duration, blood transfusions, and procedural complications (conversion to sternotomy or on-pump, bleeding, intraoperative death). Immediate postoperative outcomes included wound complications, myocardial infarction, cerebrovascular accidents, cardiac complications (atrial fibrillation, pericardial effusion), pulmonary issues (pneumothorax, pleural effusion), renal dysfunction, ventilation needs, ICU and hospital stay duration, and 30-day mortality.

Follow-up data encompassed monitoring intervals, angina, repeat revascularization, mortality, myocardial infarction and cerebrovascular events. Patients were grouped into conventional and robot-assisted HCR cohorts subsequently.

### 2.4 Statistics

Descriptive statistics summarized clinical and procedural characteristics. Continuous variables were presented as medians with interquartile ranges; categorical data as frequencies with percentages. Comparative analysis was conducted using appropriate statistical tests. A threshold of *p* < 0.05 was used to define statistical significance, indicating a low probability of the observed differences occurring due to chance. Descriptive tables provided a structured presentation of the data, enabling interpretation and analysis.

## 3 Results

### 3.1 Literature research

The PubMed search yielded 105 publications, 44 of which were excluded prior to screening for being reviews or meta-analyses. The remaining 61 were screened for eligibility based on abstracts. During this process, 34 publications were excluded: 24 did not meet the inclusion criteria; 2 presented the same patient cohort, leading to exclusion of 1; 6 did not have a full text available in English or German; 5 lacked sufficient patient data on the HCR cohort.

4 additional studies were identified from an editorial published by Michael O Kayatta et al. The Deutsches Herzzentrum der Charité (DHZC) compendium provided 10 further studies; of these, 4 met the inclusion criteria after abstract screening. The remaining 6 were excluded: 2 did not meet the criteria, 2 were duplicates of already included cohorts, and 2 lacked sufficient patient data for the HCR cohort.

Following full-text evaluation, 32 studies were included, encompassing data on a total cohort of 2,048 patients (Supplementary Material). During this evaluation, the publication by Anthony Alozie et al. required exclusion of 1 patient due to missing PCI data; the remaining 3 were included. Whilst some publications provided individual patient-level data, others reported aggregate cohort-level data.

Data quality varied greatly, which was addressed in the comparative tables by calculating the percentage of missing data values. Overall, an almost complete data set was available for patient demographics and coronary findings. However, descriptions of cardiovascular symptom severity were inconsistent, limiting the use of NYHA and CCS classification systems. Follow-up information varied in both availability and quality, with differing collection intervals complicating outcome comparisons between cohorts. Moreover, important preoperative risk scores like EuroSCORE, SYNTAX Score, Parsonnet Score or STS PROM and PROMOM Score were inconsistently and inadequately reported.

### 3.2 Patient demographics and coronary findings

A data set of 2,048 patients was obtained and subsequently grouped into robot-assisted HCR (*n* = 903) and conventional HCR (*n* = 1,145). A comparative analysis of patient demographics and clinical traits was conducted (Table 1).

**Table 1:** Patient demographics.

	Robot-assisted HCR patient cohort (*n* = 903)	Conventional HCR patient cohort (*n* = 1,145)
Age (years)	51.69 (±7.77)	69.62 (±8.42)
Sex (Male:Female)	670: 233 (74.20%): (25.80%)	890: 255 (77.73%): (22.27%)
BMI (kg/m^2^)	34.66 (±13.13)	26.62 (±1.30)
Obesity (BMI >30 kg/m^2^)	37 (4.10%)	81 (7.07%)
Hypertension	591 (65.45%)	744 (64.98%)
Dyslipidemia	362 (40.09%)	492 (42.97%)
Diabetes mellitus		
Insulin-dependent	1 (0.11%)	36 (3.14%)
Insulin-independent	259 (26.68%)	277 (24.19%)
Smoking		
Active	95 (10.52%)	322 (28.12%)
Ex-smoker	31 (14.51%)	0 (0.00%)
Never	160 (17.72%)	1 (0.09%)
NYHA classification		
NYHA I	0 (0.00%)	0 (0.00%)
NYHA II	18 (1.99%)	90 (7.86%)
NYHA III	5 (0.55%)	33 (2.88%)
NYHA IV	35 (3.88%)	22 (1.92%)
CCS classification		
CCS I	3 (0.33%)	0 (0.00%)
CCS II	47 (5.20%)	1 (0.09%)
CCS III	139 (15.39%)	20 (1.75%)
CCS IV	79 (8.75%)	24 (2.10%)
Unstable angina	6 (0.66%)	245 (21.40%)
Family history of cardiac disease	11 (1.22%)	60 (5.24)
Prior MI		
NSTEMI	259 (28.68%)	156 (13.62%)
STEMI	1 (0.11%)	23 (2.01%)
Clinical preoperative scores		
EuroSCORE	1.57 (±0.62)	6.30 (±9.84)
STS: PROM	2 (±0.00)	3.30 (±1.35)
PROMOM	—	31.59 (±0.00)
SYNTAX Score	25.00 (±14.14)	16.93 (±10.11)
Parsonnet Score		26.33 (±5.62)

Data is presented as mean ± standard deviation or absolute figures (*n*).

Patients in the robot-assisted cohort were substantially younger compared to those in the conventional cohort (51.69 ± 7.77 vs. 69.62 ± 8.42 years). A male predominance was observed in both cohorts (74.20% vs. 77.73%). While the robot-assisted cohort exhibited a higher mean BMI (34.66 ± 13.13 vs. 26.62 ± 1.30), the prevalence of obesity was greater in the conventional group (7.07% vs. 4.10%). The apparent discrepancy between the mean BMI and the proportion of patients reported as obese reflects heterogeneity in data presentation across the included studies, with some reporting BMI as a continuous variable only and others as a categorical measure of obesity.

Hypertension was a common comorbidity (65.45% in robot-assisted vs. 64.98% in conventional), as was dyslipidemia (40.09% vs. 42.97%). Insulin-dependent diabetes was more prevalent in the conventional cohort (3.14% vs. 0.11%), while insulin-independent diabetes showed a similar prevalence (24.19% vs. 26.68%). Instances where diabetes mellitus was reported without an explicit specification of insulin dependency were categorized as insulin-independent by default.

Smoking patterns differed substantially, with active smoking being more prevalent in the conventional cohort (28.12% vs. 10.52%) and non-smoking more frequent in the robot-assisted group (17.72% vs. 0.09%). Ex-smokers represented 14.51% of the robot-assisted group, whilst none were reported in the conventional group. Individuals with a “history of smoking” without further clarification were attributed to the ex-smoker category.

Clinical classifications revealed more advanced cardiac symptoms in the conventional group: severe heart failure (NYHA III or IV) affected 4.80% vs. 4.43%, and unstable angina was more prevalent (21.40% vs. 0.66%). In contrast, severe angina (CCS III or IV) was more common in the robot-assisted cohort (24.14% vs. 3.85%). The variability and ambiguity in cardiovascular symptomology descriptions across the studies included necessitated several assumptions during data collection: stable angina was attributed to CCS II, progressive angina to CCS III, mean values were rounded to the closest group, and when a classification spanned two categories the higher class was reported. The observed difference between the reported prevalence of unstable angina and CCS class III/IV symptoms reflects heterogeneity in reporting among included studies, with some classifying unstable angina as a separate diagnostic entity and others using CCS grading only for stable angina. These assumptions, while necessary to standardize data, introduced challenges in the application of NYHA and CCS classifications.

Cardiovascular history varied between the groups. A family history of cardiac disease was reported more frequently in the conventional cohort (5.24% vs. 1.22%). Non-ST-elevation myocardial infarction (NSTEMI) was more prevalent in the robot-assisted group (28.68% vs. 13.62%), whereas ST-elevation myocardial infarction (STEMI) was somewhat more common in the conventional group (2.01% vs. 0.11%). All unspecified myocardial infarctions in the data were assumed to be STEMIs, as these are more strongly associated with extensive coronary artery occlusion and the consequent need for coronary revascularization.

Preoperative risk scores showed notable differences. The robot-assisted cohort had lower surgical risk scores, namely the EuroSCORE (1.57 ± 0.62 vs. 6.30 ± 9.84) and STS PROM score (2 ± 0 vs. 3.30 ± 1.35), while it exhibited higher coronary complexity, reflected by the SYNTAX (25.00 ± 14.14 vs. 16.93 ± 10.11). The Parsonnet score was reported only for the conventional group (26.33 ± 5.62).

However, it is important to consider that these scores were only scarcely reported, and those that were provided varied significantly, with no consistency in the type of score used. One study reported the SYNTAX score, while another used the Parsonnet score. This lack of coherence makes it impossible for these scores to accurately represent the cohorts included in the review, thereby hindering the ability to draw reliable conclusions on the differing preoperative risk.

The mean preoperative left ventricular ejection fraction (EF), measured via transthoracic echocardiography (TTE), was similar in both patient cohorts (Table 2): 51.69% (±13.86) in the robot-assisted cohort and 52.50% (±9.11) in the conventional cohort (*p* = 0.192).

**Table 2:** Preoperative ejection fraction.

	Robot-assisted HCR patient cohort (*n* = 903)	Conventional HCRpatient cohort (*n* = 1,145)	*p*-value	Missing data (%)
Ejection fraction (TTE)	51.69 (±13.86)	52.50 (±9.11)	0.192	29.7
EF <35%	1 (0.11%)	62 (5.41%)	0.373	32.4
EF 35%–39%	2 (0.22%)	0 (0.00%)	0.203	35.1
EF 40%–55%	444 (49.17%)	576 (50.31%)	0.634	32.4
EF >55%	211 (23.37%)	354 (30.92%)	0.45	23.4

Data is presented as mean ± standard deviation or absolute figures (*n*).

Patients with a severely reduced EF (<35%) were rare in the robot-assisted cohort (0.11%) but more common in the conventional cohort (5.41%, *p* = 0.373). In the EF range of 35%–39%, there were very few cases in either group (0.22% in robot-assisted vs. 0.00% in conventional, *p* = 0.203). Moderate EF (40%–55%) was similarly distributed in both cohorts, (49.17% vs. 50.31%, *p* = 0.634), as was EF >55% (23.37% vs. 30.92%, *p* = 0.45).

The percentage of missing data for each category was also noted, ranging from 23.4% to 35.1%, which reflects the proportion of patients for whom preoperative EF measurements were unavailable or not recorded. This level of missing data should be considered when interpreting the findings, as it may impact the robustness of comparisons between the cohorts.

### 3.3 Coronary findings and procedural details

Preoperative coronary angiographic findings (Table 3) predominantly described culprit LAD lesions where the exact location of the stenosis was not further specified (categorized as “unspecified”): 96.90% in the robotic and 96.59% in the conventional cohort (*p* = 0.994). Only a limited number of studies provided a more detailed location. Proximal LAD lesions were more frequent in the conventional cohort (3.23% vs. 1.11%, *p* = 0.392), while mid LAD lesions and distal LAD lesions were described exclusively in the robot-assisted cohort, (0.33%, *p* = 0.258 and 1.66%, *p* = 0.309). The percentage of missing data for all specific LAD locations was 21.6%, whilst 13.5% were missing for the unspecified LAD category.

**Table 3:** Coronary findings.

	Robot-assisted HCR patient cohort (*n* = 903)	Conventional HCR patient cohort (*n* = 1,145)	*p*-value	Missing data (%)
Culprit lesion				
Proximal LAD	10 (1.11%)	37 (3.23%)	0.392	21.6
Mid LAD	3 (0.33%)	0 (0.00%)	0.258	21.6
Distal LAD	15 (1.66%)	0 (0.00%)	0.309	21.6
LAD (unspecified)	875 (96.90%)	1,106 (96.59%)	0.994	13.5
Other stenotic lesions				
Right Coronary Artery (RCA)	168 (18.60%)	184 (16.07%)	0.004	56.8
Diagonal Branch 1 (D1)	22 (2.44%)	7 (0.61%)	0.208	54.1
Obtuse Marginal Artery of the	36 (3.99%)	1 (0.09%)	0.628	54.1
Circumflex Artery (OM)				
Left Circumflex Artery (LCx)	91 (10.08%)	168 (14.67%)	0.745	56.8
Left Main (LM)	11 (1.22%)	33 (2.88%)	0.709	56.8

Data is presented as mean ± standard deviation or absolute figures (*n*).

Culprit LAD lesions were revascularized with a MIDCAB procedure consisting of a LIMA-LAD anastomosis. Among the 14 studies included for the robot-assisted cohort, 4 studies (Torregrossa et al.; Aerden et al.; Lee et al.; Halkos et al.) reported an off-pump procedure. In the conventional cohort 3 out of 18 studies (Repossini et al.; Modrau et al.; Torre et al.) described off-pump MIDCAB procedures. Notably, in the conventional cohort, the study by Modrau et al. utilized an inferior reversed J-hemisternotomy approach, while Dullum et al. described a xiphoid technique, where the procedure was performed through an incision over the xiphoid.

PCI was performed on target lesions of non-LAD vessels. Stenotic lesions in the right coronary artery (RCA) were found to be significantly more frequent in the robot-assisted cohort (18.60% vs. 16.07%, *p* = 0.004). However, this finding is complicated by the high percentage of missing data, which stands at 56.8% for RCA stenosis. This means that for more than half of the patients in the study, there was no data available for this specific category, significantly limiting the completeness of the analysis.

Stenosis of both the diagonal branch (D1) and the obtuse marginal artery (OM) was nominally more frequent in the robot-assisted cohort (2.44% vs. 0.61%, *p* = 0.208 and 3.99% vs. 0.09%, *p* = 0.628, respectively). Circumflex artery lesions (LCx) were slightly more common in the conventional cohort (14.67% vs. 10.08%, *p* = 0.745), as was left main artery (LM) stenosis (1.22% vs. 2.88%, *p* = 0.709). The percentage of missing data for the remaining stenotic lesions was also notably high, ranging from 54.1% to 56.8%. This level of missing data highlights potential limitations in the completeness and reliability of these findings.

### 3.4 Interventional details and procedural complications

The PCI and MIDCAB components of the HCR procedure can be performed in a simultaneous approach, where PCI is performed intraoperatively, or in a staged approach, where PCI is scheduled either pre- or postoperatively (Table 4). In this study, the proportion of patients undergoing postoperative PCI was higher in the robot-assisted group (45.29% vs. 35.81%, *p* = 0.901), with an average timing of 23.50 (±34.64) vs. 5.65 days (±8.66, *p* = 0.158).

**Table 4:** PCI timing & stent placement.

	Robot-assisted HCR patient cohort (*n* = 903)	Conventional HCR patient cohort (*n* = 1,145)	*p*-value	Missing data (%)
Postoperative PCI	409 (45.29%)	410 (35.81%)	0.901	25
Timing (Days)	23.50 (±34.64)	5.65 (±8.66)	0.158	52.8
Preoperative PCI	96 (10.63%)	386 (33.71%)	0.23	25
Timing (Days)	11.13 (±20.94)	29.14 (±41.05)	0.219	75
Intraoperative PCI	204 (22.59%)	5 (0.44%)	0.11	25
Timing (Days)	0.00	0.00	—	—
Stents				
Right Coronary Artery (RCA)	293 (32.45%)	305 (26.64%)	0.359	43.2
Diagonal Branch 1 (D1)	43 (4.76%)	11 (0.96%)	0.292	43.2
Obtuse Marginal Artery of the	26 (2.88%)	137 (11.97%)	0.666	45.9
Circumflex Artery (OM)				
Left Circumflex Artery (LCx)	283 (31.34%)	131 (11.44%)	0.671	43.2
Left Main (LM)	9 (1.00%)	10 (0.87%)	0.737	48.6
Stent typology				
Bare metal stents	81 (8.97%)	109 (9.52%)	0.643	81.1
Drug-eluting stents	566 (62.68%)	62 (5.42%)	0.027	73

Data is presented as mean ± standard deviation or absolute figures (*n*).

Preoperative PCI was more frequent in the conventional cohort (33.71% vs. 10.63%, *p* = 0.23), with a longer average timing (29.14 ± 41.05 vs. 11.13 ± 20.94 days, *p* = 0.219). However, for both the preoperative and postoperative PCI categories, the missing data percentages for the timing are alarmingly high, with 52.8% missing data for postoperative PCI and 75% for preoperative PCI. This may skew the results and limit their generalizability when it comes to comparing the timing of PCI.

Intraoperative PCI was markedly more frequent in the robot-assisted cohort (22.59% vs. 0.44%, *p* = 0.11). The percentage of missing data for intraoperative PCI (25%) is relatively low and consistent with the missing data rates for pre- and postoperative PCI (both also 25%). Since the timing categories for PCI are mutually exclusive, the identical missing percentage across all categories reflects studies where details on the staging or simultaneity of PCI and MIDCAB were entirely absent.

Stent typology data (Table 4) highlighted a significant difference in the use of drug-eluting stents, which were more common in the robot-assisted cohort (62.68% vs. 5.42%, *p* = 0.027). Bare metal stents were used at similar rates between the groups (8.97% vs. 9.52%, *p* = 0.643).

The high percentage of missing data in this category, particularly for stent typology (81.1% and 73%), undermines the reliability and interpretability of these findings, as it raises concerns about selection bias. While the existing data suggests a notable discrepancy in drug-eluting stent usage, the extent of missing information precludes firm conclusions, underscoring the need for more comprehensive data to ensure validity.

Operative time was longer in the robot-assisted cohort (209.67 ± 81.65 vs. 149.33 ± 38.04 min) (Table 5). Blood transfusions were more frequently required in the conventional cohort (10.83% vs.4.65%; *p* = 0.85). Conversions to sternotomy exhibited a slightly higher incidence in the robot-assisted group (1.77% vs. 0.96%, *p* = 0.255) and was further complicated by 40.5% missing data. Conversions to on-pump surgery were infrequent, (0.11% vs. 0%, *p* = 0.23; 45.9% of missing data), and the incidence of revision for bleeding was marginally higher in the robot-assisted group (2.44% vs. 1.48%, *p* = 0.654; 35.1% of data missing).

**Table 5:** Procedural complications.

	Robot-assisted HCR patient cohort (*n* = 903)	Conventional HCR patient cohort (*n* = 1,145)	*p*-value	Missing data (%)
Operative time (min)	209.67 (±81.65)	149.33 (±38.04)	—	—
Blood transfusions	42 (4.65%)	124 (10.83%)	0.85	51.4
Conversion to sternotomy	16 (1.77%)	11 (0.96%)	0.255	40.5
Conversion to on-pump	1 (0.11%)	0 (0.00%)	0.23	45.9
Revision for bleeding	22 (2.44%)	17 (1.48%)	0.654	35.1
Intraoperative death	0 (0.00%)	1 (0.09%)	0.362	24.3

Data is presented as mean ± standard deviation or absolute figures (*n*).

Intraoperative mortality was a rare event, with no cases in the robot-assisted cohort and one (0.09%) in the conventional group, (*p* = 0.362), though 24.3% of data were missing, further limiting the conclusiveness of this outcome.

### 3.5 Outcomes and follow-up

Wound complications were more frequent in the robot-assisted cohort (0.66% vs. 0.09%, *p* = 0.498), while major adverse cardiovascular and cerebrovascular events (MACCE) were only reported in the conventional group (0.79%, *p* = 0.126) (Table 6). The incidence of myocardial infarction (0.78% vs. 1.14%, *p* = 0.227), cerebrovascular accidents (0.89% vs. 0.26%, *p* = 0.555), and atrial fibrillation (11.96% vs. 9.96%, *p* = 0.966) did not differ significantly between the two cohorts. Pneumothorax and pleural effusion were more frequently observed in the conventional HCR group (1.83% vs. 0.11%, *p* = 0.375 and 10.74% vs. 0.00%, respectively; *p* = 0.144). Pericardial effusion (0.61% vs. 0.11%, *p* = 0.756) and renal dysfunction (0.61% vs. 0.11%, *p* = 0.641) showed no significant differences.

**Table 6:** Postoperative course and follow-up.

	Robot-assisted HCR patient cohort (*n* = 903)	Conventional HCR patient cohort (*n* = 1,145)	*p*-value	Missing data (%)
Postoperative course				
Wound complications	6 (0.66%)	1 (0.09%)	0.498	35.1
MACCE^a^	0 (0.00%)	9 (0.79%)	0.126	35.1
Myocardial infarction	7 (0.78%)	13 (1.14%)	0.227	29.7
Cerebrovascular accident	8 (0.89%)	3 (0.26%)	0.555	29.7
Atrial fibrillation	108 (11.96%)	114 (9.96%)	0.966	27
Pneumothorax	1 (0.11%)	21 (1.83%)	0.375	35.1
Pleural effusion	0 (0.00%)	123 (10.74%)	0.144	35.1
Pericardial effusion	1 (0.11%)	7 (0.61%)	0.756	35.1
Renal dysfunction	1 (0.11%)	7 (0.61%)	0.641	27
30-day mortality	4 (0.44%)	8 (0.70%)	0.239	18.9
Postoperative ventilation (h)	6.57 (±6.21)	7.01 (±5.60)	0.848	62.2
No. patients >24 h	17 (1.88%)	8 (0.70%)	0.316	81.1
ICU stay (h)	23.50 (±1.00)	17.95 (±10.89)	0.127	70.3
Discharge (post-op day)	4.27 (±1.34)	10.27 (±7.34)	0.001	37.8
Follow-up				
Follow-up timing (months)	23.42 (±24.36)	40.21 (±38.76)	0.322	18.9
Angina episodes	50 (5.54%)	41 (3.58%)		
LIMA-LAD revascularization	26 (2.88%)	38 (3.32%)		
Non-LAD lesions	42 (4.65%)	51 (4.45%)		
Revascularization				
Mortality	24 (2.66%)	54 (4.72%)		
Myocardial infarction	7 (0.78%)	43 (3.76%)		
Stroke	4 (0.44%)	6 (0.52%)		

Data is presented as mean ± standard deviation or absolute figures (*n*).

a Some studies reported MACCE only, some detailed myocardial infarction and cerebrovascular events, while some also reported both.

Thirty-day mortality was low and comparable (0.44% vs. 0.70%, *p* = 0.239). Postoperative ventilation duration was similar (6.57 vs. 7.01 h, *p* = 0.848), and prolonged ventilation (>24 h) was more frequent in the robot-assisted group (1.88% vs. 0.70%, *p* = 0.316). However, both variables had high percentages of missing data (62.2% and 81.1%, respectively), thus limiting the reliability of these findings. ICU stay was longer in the robot-assisted cohort (23.50 vs. 17.95 h, *p* = 0.127), though a substantial proportion of data was again missing (70.3%).

A significant difference was observed in hospital length of stay, with earlier discharges in the robot-assisted cohort (4.27 vs. 10.27 postoperative days, *p* = 0.001). However, missing data for this metric (37.8%) should be considered when interpreting these results.

In this review, the mean follow-up duration differed substantially between the two patient cohorts, with those undergoing robot-assisted HCR experiencing a markedly shorter follow-up period compared to their counterparts in the conventional HCR group (23.42 ± 24.36 vs. 40.21 ± 38.76 months, *p* = 0.322). Data on the timing of follow-up was available for the majority of patients, with missing data amounting to only 18.9%, indicating a relatively high level of completeness. However, the substantial variability in follow-up duration between patients, as indicated by the high standard deviations in both cohorts, limits the strength of conclusions regarding comparative long-term outcomes.

The conventional HCR cohort demonstrated higher rates of several adverse outcomes compared to the robot-assisted group. Mortality was higher in the conventional cohort (4.72% vs. 2.66%), as were the incidences of myocardial infarction (3.76% vs. 0.78%) and stroke (0.52% vs. 0.44%). In contrast, recurrent angina was reported more frequently in the robot-assisted cohort (5.54% vs. 3.58%). With respect to revascularization outcomes, the need for LIMA-to-LAD reintervention was slightly higher in the conventional cohort (3.32% vs. 2.88%), while non-LAD lesion revascularization rates were largely comparable between the two groups (4.45% in conventional HCR vs. 4.65% in robot-assisted HCR).

## 4 Discussion

**Figure 2:**
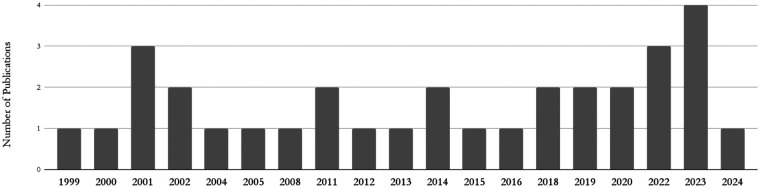
Number of included publications by year.

Over the past two decades, HCR has gained increasing clinical and academic interest, reflecting a broader shift in cardiovascular surgery toward minimally invasive, patient-centered strategies. As depicted by the 32 studies included in this systematic review (Figure 2), HCR-related literature has steadily grown over the past 25 years, emphasizing the growing role of HCR within contemporary revascularization practices. This growing implementation likely stems from technological advancements, evolving clinical guidelines, and enhanced emphasis on reducing invasiveness without compromising efficacy (16, 17, 18).

HCR was first formally recognized in the 2011 ACCF/AHA guidelines (20), later receiving a Class IIb recommendation in the 2014 (21) and 2018 (22) ESC/EACTS guidelines for selected patients with multivessel CAD. The 2018 update elevated the level of supporting evidence from C to B, reflecting accumulating clinical data and institutional expertise in high-volume multidisciplinary centers. Dokollari et al. reported a substantial increase in HCR adoption within their institution, with hybrid procedures among robotic CABG cases rising from 25.5% in 2005–2010 to 48.4% in 2017–2021 (23).

Nevertheless, HCR's real-world utilization has remained limited, as reflected by the small cohort of this study. The Society of Thoracic Surgeons (STS) database shows that between 2011 and 2013, HCR accounted for only 0.48% of all CABG procedures (24). A decline in HCR literature in 2024 (Figure 2) may partly result from delayed procedural backlogs due to the COVID-19 pandemic (25, 26). Additionally, complex data protection regulations like the GDPR raise administrative and ethical barriers to publishing individual patient data (27, 28).

Demographic patterns within robot-assisted HCR cohorts reveal a distinctly lower-risk cardiovascular profile. Patients undergoing robotic procedures were, on average, two decades younger than those undergoing conventional HCR (51.69 ± 7.77 vs. 69.62 ± 8.42 years), with lower rates of insulin-dependent diabetes (0.11% vs. 3.14%), smoking (10.52% vs. 28.12%), and family history of cardiac disease (1.22% vs. 5.24%). Despite a higher mean BMI, obesity (BMI >30 kg/m^2^) was less prevalent in the robotic cohort, suggesting that body composition was a factor in determining surgical eligibility.

Myocardial infarction patterns support this selective approach: NSTEMI predominated in the robotic cohort, while STEMI, often associated with urgent presentations, occurred more frequently in the conventional group. This suggests that robotic HCR was reserved for stable subacute coronary syndromes, while conventional HCR addressed acute or high-risk pathology. These findings reflect the selection bias in robotic programs, which favor fewer comorbidities and higher surgical eligibility (3, 16, 17), consistent with reports of robotic HCR mostly being performed in elective, lower-risk patients by specialized cardiovascular teams (29).

The higher SYNTAX scores observed in the robotic group imply greater coronary complexity and, coupled with lower systematic risk, suggest a sophisticated interplay between coronary anatomy and overall operative fitness in patient selection. However, inconsistent preoperative risk score reporting across studies weakens this inference. While EuroSCORE II and STS PROM were lower in the robotic cohort, inconsistent and incomplete data reporting limits their representativeness. The Parsonnet score was reported only in the conventional group, thus preventing cross-cohort comparison. This lack of standardized, uniform risk profiling complicates definitive conclusions about procedural impact on outcomes.

The trend toward younger, lower-risk patients in robotic HCR aligns closely with established selection guidelines, which recommend HCR in patients with suitable LAD lesions for bypass and non-LAD lesions for PCI (1, 2), ideally those with low-to-intermediate SYNTAX scores and comorbidities that heighten surgical risk (30, 31). Robotic HCR requires stable hemodynamics, suitable anatomy, and absence of contraindications like intramyocardial LAD targets or prior thoracic surgery (16, 17, 32). The observed patient profile aligns with these prerequisites, whereas the conventional HCR group included more acutely ill and comorbid patients, reflecting its greater suitability for urgent or anatomically complex cases.

Anatomical distribution of non-LAD lesions further reflects procedural selection guided by lesion complexity. While not statistically significant, lesions in the diagonal and obtuse marginal branches were more frequent in the robot-assisted cohort, whereas circumflex and left main disease predominated in the conventional group. Right coronary artery stenosis was significantly more frequent in the robotic cohort (*p* = 0.004), aligning with its lower SYNTAX score (12) due to a more linear trajectory, fewer bifurcations, and a smaller perfused myocardial territory, rendering them more amenable to straightforward PCI (33). This increased RCA disease within the robotic cohort aligns with the broader trend of selecting patients with simpler coronary anatomy and lower procedural complexity.

PCI timing differences between cohorts likely reflect institutional logistics, patient selection, and procedural infrastructure. Robotic HCR more frequently performed postoperative PCI with longer surgery-to-PCI intervals, while in conventional HCR preoperative PCI prevailed. Intraoperative PCI, requiring real-time coordination and specialized hybrid facilities, was markedly more common in the robotic cohort, consistent with the infrastructural advantages and procedural integration inherent to robotic programs. These trends may reflect the clinical rationale: CABG-first is preferred when the LAD is the culprit lesion in ACS or presents critical stenosis in stable angina, supporting simultaneous or postoperative PCI in HCR (11, 30, 31). Conversely, PCI-first is favored in ACS arising from non-LAD lesions or stable patients with critical non-LAD disease, allowing stabilization before CABG, which is often delayed to mitigate perioperative bleeding risks from dual antiplatelet therapy and residual anticoagulation (34, 35).

The significantly increased use of drug-eluting stents (DES) in the robot-assisted cohort, further reinforces the association of robotic HCR and technologically advanced, guideline-concordant interventional practices. DES offer superior long-term patency and reduced restenosis rates than bare-metal stents (BMS) (8, 36), and their adoption has become standard in elective PCI settings with manageable bleeding risk and anatomical complexity (37, 38). Their preference in robotic cohorts may reflect access to advanced tools and an emphasis on long-term outcomes in centers specializing in minimally invasive hybrid approaches. However, PCI timing, whether pre-, intra-, or postoperative, and stent typology, whether DES or BMS, can significantly affect outcomes, particularly bleeding, reintervention, and MACCE. While staged HCR may reduce bleeding and repeat procedures, single-stage approaches show lower MACCE rates (39). Given the procedural variability, more targeted comparative studies are needed to clarify benefits and risks across HCR strategies.

Analysis of procedural complications reveals notable differences. Robotic HCR demonstrated considerably longer mean operative timing (209.67 ± 81.65 vs. 149.33 ± 38.04 min), with substantial standard deviation likely reflecting operator experience, case complexity, and the steep learning curve inherent to robotic surgery. Previous studies indicate that 50–100 cases are required for proficiency (40), with significant improvements after 15–50 cases (41, 42). These findings support centralizing robotic HCR in high-volume centers with dedicated training programs (34, 43, 44).

Despite extended procedural times, robotic HCR experienced a lower incidence of intraoperative blood transfusions (4.65% vs. 10.83%), consistent with evidence highlighting reduced bleeding as a key benefit of minimally invasive HCR (4, 11, 45–47). Enhanced visualization, limited dissection, and sternotomy avoidance contribute to this outcome, though missing data (51.4%) may limit interpretation.

Marginally higher rates of conversion to sternotomy (1.77% vs. 0.96%), conversion to on-pump surgery (0.11% vs. 0%), and revision for bleeding (2.44% vs. 1.48%) in the robotic group, while not statistically significant, likely reflect procedural complexity and the need for greater surgical expertise. This underscores the steep learning curve associated with robot-assisted MIDCAB (17, 48, 49) and reinforces the importance of structured proctoring and simulation-based training during early adoption (34, 44).

Intraoperative mortality was rare (0% in robotic vs. 0.09% in conventional), consistent with literature affirming the safety of both HCR strategies when applied to appropriately selected patients (50, 51). Avoiding sternotomy reduces deep sternal wound infection risk (4, 11), especially relevant in patients with diabetes or obesity (34). Furthermore, off-pump techniques, performed in 4 robotic and 3 conventional studies, may enhance safety by eliminating the risks associated with cardiopulmonary bypass (CPB), including systematic inflammation and end-organ dysfunction (4, 34).

Comparable postoperative complication rates between cohorts reinforces that robotic assistance does not compromise procedural safety, despite its technical complexity. Similar incidence of myocardial infarction, stroke, atrial fibrillation, and renal dysfunction support the short-term safety of minimally invasive approaches (16–18, 50, 51). Unexpected for minimally invasive techniques (52), wound complications were slightly more frequent in the robot-assisted cohort, possibly due to underreporting or early learning-curve cases. Conversely, pleural complications were more frequent in the conventional cohort, reflecting the more invasive thoracic exposure. ICU stay and ventilation time were longer in robotic HCR, though interpretation is limited by extensive missing data. These differences may reflect longer operative times or cautious postoperative monitoring. Nonetheless, the low 30-day mortality in both cohorts confirms the overall safety of HCR.

The significantly shorter hospital length of stay in the robot-assisted cohort (4.27 vs. 10.27 days, *p* = 0.001) is a finding with meaningful clinical and economic implications. Earlier discharge likely reflects smaller incisions, reduced postoperative pain, and faster mobilization, aligning with broader evidence linking robotic surgery to accelerated recovery and lower resource utilization (11, 46, 47, 53). From an economic perspective, a shorter hospital stay directly reduces perioperative resource consumptions and overall inpatient cost, potentially offsetting higher intraoperative expenses related to robotic equipment and set up. While studies in conventional coronary surgery have shown that less invasive and off-pump techniques reduce material use and transfusion requirements (14, 15), Halkos et al. demonstrated that, despite higher fixed procedural costs, hybrid revascularization achieved comparable variable costs and greater hospital contribution margins through improved postoperative efficiency (18). Earlier discharge may improve patient satisfaction, reduce nosocomial risks, and ease hospital burden, making robotic HCR attractive from a healthcare system's perspective. However, staged procedures may mask total hospitalization time. The shorter postoperative stays support robotic HCR's potential to enhance safety, recovery, and efficiency in high-volume centers (34, 44).

Evaluating long-term outcomes requires consistent follow-up. The markedly shorter mean follow-up duration in the robot-assisted cohort (23.42 ± 24.36 vs. 40.21 ± 38.76 months) limits detection of late adverse events and introduces temporal bias that challenges the reliability of comparative conclusions. Despite 81.1% data completeness, inconsistent follow-up prevents robust, time-adjusted comparisons and likely reflects differences in surveillance duration rather than procedural efficacy.

Higher rates of mortality, myocardial infarction, and stroke in the conventional cohort may stem from longer follow-up rather than procedural inferiority. The robotic cohort's advantage may reflect the inherent selection of lower-risk, electively treated patients in specialized centers with multidisciplinary expertise, a pattern well documented in previous studies (29). As a recent development (16, 18), robotic HCR lacks long-term follow-up data. However, emerging mid-term results are encouraging. Adams et al. reported 91% survival, 94% angina-free status, and 87% freedom from reintervention at five years in single-stage robotic HCR (51). Bonatti et al. observed 92.9% five-year survival and 75.2% MACCE-free survival (50), while Davidavicius et al. reported no major adverse events over 19 months (54). These findings suggest robotic HCR may provide durable outcomes in well-selected populations, although further multicenter validation remains necessary.

Recurrent angina, even though not statistically significant, was slightly more common in robotic HCR (5.54% vs. 3.58%). Despite the shorter follow-up, this could reflect incomplete revascularization or differences in lesion complexity and anatomical targeting. The SYNTAX and EXCEL trials show that ischemia tends to recur in different territories depending on revascularization strategy: LAD following PCI, and non-LAD following CABG (12, 55, 56). In HCR, this highlights the importance of durable LIMA-LAD grafting and precise PCI targeting. More intensive monitoring in robotic cases may also explain the increased symptom detection.

Reintervention rates were low and comparable. LIMA-to-LAD reintervention was slightly more frequent in the conventional group (3.32% vs. 2.88%), with identical non-LAD revascularization rates, suggesting comparable procedural durability. However, conclusions remain limited by follow-up disparities. Definitive long-term efficacy comparisons will require prospective studies with harmonized follow-up, standardized outcomes, and adequate statistical power.

## 5 Limitations

This systematic review and meta-analysis has several methodological limitations. A major concern is the high degree of missing data across clinical and procedural variables, impairing overall integrity and comparability. The inconsistent or non-utilization of standardized preoperative risk scores (EuroSCORE, SYNTAX, STS PROM, Parsonnet) represents a critical shortcoming. More consistent application of these scores, especially the SYNTAX score, would clarify the influence of coronary complexity on surgical strategy. Similarly, stricter and standardized application of NYHA and CCS classifications is needed to accurately reflect functional status and symptom burden. The sporadic application limits insight into whether outcomes reflect surgical technique or underlying patient risk.

Data source heterogeneity further compounds this issue. Some studies contributed individual-level data, whereas others offered only aggregated statistics, limiting the granularity of risk-adjusted comparisons. Without traceable patient-specific outcomes, variations in patient selection, procedural execution, and postoperative care may be obscured, undermining generalizability of the review's findings.

Economic parameters were not reported in any of the included studies, preventing a quantitative comparison of procedural or hospital costs. Essential cost components, such as intraoperative material expenditure, operative time, disposable instrumentation, and postoperative resource utilization, were inconsistently or not documented, precluding meaningful evaluation of the financial impact of robotic vs. conventional hybrid revascularization. Consequently, any economic inference drawn from indicators such as length of stay or transfusion rates remains speculative. To determine the true value of robotic-assisted HCR within a global cost–benefit framework, future investigations must incorporate standardized, comprehensive cost analyses encompassing fixed and variable expenditures, reimbursement structures, and long-term healthcare resource utilization.

Unequal follow-up duration presents another limitation. The robot-assisted cohort had a considerably shorter mean follow-up (23.4 ± 24.4 vs. 40.2 ± 38.8 months), introducing temporal bias in assessing long-term outcomes such as mortality, myocardial infarction, stroke, recurrent angina, and revascularization. A shorter surveillance window may underestimate late adverse events, potentially creating a misleading impression of procedural superiority.

Wide variation in follow-up durations within and across cohorts further hampers comparability. Stricter standardized follow-up intervals (e.g., 6 months, 1 year, 5 years, 10 years) would improve identification of time-dependent complications and facilitate survival and event-free curve analyses.

Finally, incomplete reporting of PCI timing relative to MIDCAB was frequent, with substantial missing data (52.8% postoperative, 75% preoperative). Without precise PCI-to-MIDCAB intervals, it remains difficult to assess how timing influences procedural success, complication rates, and long-term outcomes. These findings highlight the need for a prospective, multicenter registry with standardized reporting.

## 6 Conclusion

This pooled analysis of 2,048 patients undergoing hybrid coronary revascularization suggests robotic assistance may offer perioperative advantages. However, significant limitations necessitate cautious interpretation. Robust prospective trials with standardized reporting and long-term follow-up are essential to determine the true clinical value of robotic HCR.

## Data Availability

The original contributions presented in the study are included in the article/Supplementary Material, further inquiries can be directed to the corresponding author.

## Glossary

ACCF/AHA: American College of Cardiology Foundation/American Heart Association
ACS: acute coronary syndrome
BMI: body mass index
BMS: bare metal stents
CABG: coronary artery bypass grafting
CAD: coronary artery disease
CCS: Canadian Cardiovascular Society
CPB: cardiopulmonary bypass
D1: First diagonal branch of left anterior descending coronary artery
DES: drug-eluting stent
DHZC: Deutsches Herzzentrum der Charité
EACTS: European Association for Cardio-Thoracic Surgery
EF: ejection fraction
ESC: European Society of Cardiology
EuroScore: European System for Cardiac Operative Risk Evaluation
EXCEL: Evaluation of XIENCE vs. Coronary Artery Bypass Surgery for Effectiveness of Left Main Revascularization
GDPR: General Data Protection Regulation
HCR: Hybrid Coronary Revascularization
ICU: Intensive Care Unit
LAD: Left Anterior Descending Coronary Artery
LCx: Left Circumflex Coronary Artery
LIMA: Left Internal Mammary Artery
LM: Left Main Coronary Artery
LVAD: Left Ventricular Assist Device
MACCE: Major Adverse Cardiac and Cerebrovascular Events
MIDCAB: Minimally Invasive Direct Coronary Artery Bypass
MI: Myocardial Infarction
MCS: Mechanical Circulatory Support
NSTEMI: Non-ST-Elevation Myocardial Infarction
NYHA: New York Heart Association
OM: Obtuse Marginal Branch of the Circumflex Artery
PCI: Percutaneous Coronary Intervention
PRISMA: Preferred Reporting Items for Systematic Reviews and Meta-Analyses
PROM: Predicted Risk of Mortality
RCA: Right Coronary Artery
SVG: Saphenous Vein Graft
STEMI: ST-Elevation Myocardial Infarction
STS: Society of Thoracic Surgeons
SYNTAX: SYNergy between PCI with TAXUS and Cardiac Surgery (Score)
TTE: Transthoracic Echocardiography
