# Supplementary material for: Conventional hybrid coronary vs. robot-assisted minimally invasive direct revascularization: a meta-analysis and systematic review
Source: Front Cardiovasc Med. 2025 Dec 1;12:1650138. doi: 10.3389/fcvm.2025.1650138 (PMC12702846; doi:10.3389/fcvm.2025.1650138)
Supplement: Supplementary file 1 [file Table1.pdf]

SUPPLEMENTARY MATERIAL: Included publications and respective patient cohort

PMID	*Author*	*Title*	*Journal*	*Publication date*	*Relative HCR cohort*	*Absolute patient cohort*
INDIVIDUAL PATIENT-LEVEL DATA PROVIDED						
32459073	Alberto Repossini et al	MIDCAB Tips and tricks for a successful procedure	Multimed Man Cardiothoracic Surg.	2020	**1**	
36331815	Alexander Aerden et al	Robotic-assisted MIDCAB procedure through a minithoracotomy: Step-by-step instructions	Multimed Man Cardiothoracic Surg.	2022	**1**	
37706377	Anthony Alozie et al	Minimally invasive direct coronary artery bypass and percutaneous coronary intervention followed by transcatheter aortic valve implantation: A promising concept in high-risk octogenarians	Ann Card Anaesth	2023	**3**	***4***
12472384	Atsushi Morishita et al	Minimally invasive direct redo coronary artery bypass grafting	Ann Thorac Cardiovasc Surg.	2002	**3**	**7**
35690894	Dario T Bertolone et al	First report of totally robot-assisted hybrid coronary artery revascularization combining RE-MIDCAB and R-PCI: Case report	J Card Surg.	2022	**1**	
36872581	Gianluca Torregrossa et al	Urgent Combination of Robotic and MIDCAB Coronary Revascularization in a Morbidly Obese Patient	Innovations (Phila)	2023	**1**	
15282418	Michael S Lee et al	Hybrid revascularization using percutaneous coronary intervention and robot-assisted minimally invasive direct coronary artery bypass surgery	J Invasive Cardiol	2004	***6***	
11276464	M K Dullum et al	Xyphoid MIDCAB: report of the technique and experience with a less invasive MIDCAB procedure	Heart Surg. Forum	1999	**3**	
AGGREGATE COHORT-LEVEL DATA PROVIDED						
23444407	Alberto Repossini et al	Hybrid Revascularization in multivessel coronary artery disease	Eur J Cardiothorac Surg.	2013	**166**	
30739774	Alberto Repossini et al	Minimally invasive coronary artery bypass: Twenty-year experience	J Thorac Cardiovasc Surg.	2019	**197**	**1,060**
29517516	Alberto Repossini et al	Hybrdi coronary revascularization versus percutaneous strategies in left main stenosis: a propensity match study	J Cardiovasc Med (Hagerstown)	2018	**67**	**175**
36802970	Anne R de Jong et al	A Nationwide Study of Clinical Outcomes After Robot-Assisted Coronary Artery Bypass Surgery and Hybrid Revascularization in the Netherlands	Innovations (Phila)	2023	**91**	**440**
23956269	Corey Adams et al	Single-stage hybrid coronary revascularization with long-term follow-up	Eur J Cardiothorac Surg.	2014	**96**	
19021994	David M Holzhey et al	Minimally invasive hybrid coronary artery revascularization	Ann Thorac Surg.	2008	**117**	
11579343	D de Cannière et al	Combination of minimally invasive coronary bypass and percutaneous transluminal coronary angioplasty in the treatment of double-vessel coronary disease: Two-year follow-up of a new hybrid procedure compared with "on-pump" double bypass grafting	Am Heart J.	2001	**20**	
16159838	Giedrius Davidavicius et al	Hybrid revascularization strategy: a pilot study on the association of robotically enhanced minimally invasive direct coronary artery bypass surgery and fractional-flow-reserve-guided percutaneous coronary intervention	Circulation.	2005	**20**	
26432721	Ivy S Modrau et al	One-year clinical and angiographic results of hybrid coronary revascularization	J Thorac Cardiovasc Surg.	2015	**100**	
23103003	Johannes O Bonatti et al	Hybrid coronary revascularization using robotic totally endoscopic surgery: perioperative outcomes and 5-year results	Ann Thorac Surg.	2012	**140**	
36929299	Krzysztof Sanetra et al	Safety and feasibility of minimally invasive coronary artery bypass surgery early after drug-eluting stent implantation due to acute coronary syndrome	Kardiol Pol.	2023	**115**	

12142196	Marek Cisowski et al	Integrated minimally invasive direct coronary artery bypass grafting and angioplasty for coronary artery revascularization	Eur J Cardiothorac Surg.	2002	**50**	
24140212	Michael E Halkos et al	Clinical and angiographic results after hybrid coronary revascularization	Ann Thorac Surg.	2014	**300**	
22000276	Michael E Halkos et al	Hybrid Coronary Revascularization Versus Off-Pump Coronary Artery Bypass for the Treatment of Left Main Coronary Stenosis	Ann Thorac Surg.	2011	**27**	**108**
21939958	Michael E Halkos et al	Hybrid Coronary Revascularization Versus Off-Pump Coronary Artery Bypass Grafting for the Treatment of Multivessel Coronary Artery Disease	Ann Thorac Surg.	2011	**147**	**735**
11392640	P Presbitero et al	"Hybrid" percutaneous and surgical coronary revascularization: selection criteria from a single-center experience	Ital Heart J.	2001	**42**	
11231661	R Gao et al	"Hybrid" revascularization: video-thoracoscopy assisted MIDCAB combined with angioplasty	J Invasive Cardiol	2001	**4**	
38793110	Tiziano Torre et al	Minimally Invasive Isolated and Hybrid Surgical Revascularization for Multivessel Coronary Disease: A Single-Center Long-Term Follow-Up	J Pers Med.	2024	**14**	**92**
11145404	T Wittwer et al	Follow-up experience with coronary hybrid-revascularisation	Thorac Cardiovasc Surg.	2000	**35**	
29571730	Vincenzo Giambruno et al	Hybrid Coronary Revascularization Versus On-Pump Coronary Artery Bypass Grafting	Ann Thorac Surg.	2018	**144**	**829**
31969796	Vladimir Ganyukov et al	Randomized Clinical Trial of Surgical vs. Percutaneous vs. Hybrid Revascularization in Multivessel Coronary Artery Disease: Residual Myocardial Ischemia and Clinical Outcomes at One Year-Hybrid coronary REvascularization Versus Stenting or Surgery (HREVS)	J Interv Cardiol.	2020	**52**	**155**
27076941	Wenhui Gong et al	Robot-assisted coronary artery bypass grafting improves short-term outcomes compared with minimally invasive direct coronary artery bypass grafting	J Thorac Dis.	2016	**12**	**132**
35106840	Xibao Shi et al	Minimally invasive direct coronary artery bypass after percutaneous coronary intervention	J Card Surg.	2022	**51**	**197**
30842359	Yuki Endo et al	The Utility of a 3D Endoscope and Robot-Assisted System for MIDCAB	Ann Thorac Cardiovasc Surg.	2019	**22**	**53**
				**TOTAL COHORT:**	**2,048.00**	
